# Numerical Simulation and Experimental Study on Compound Casting of Layered Aluminum Matrix Composite Brake Drum

**DOI:** 10.3390/ma14061412

**Published:** 2021-03-15

**Authors:** Hansen Zheng, Zhifeng Zhang, Yuelong Bai

**Affiliations:** 1 National Engineering & Technology Research Center for Non-Ferrous Metal Matrix Composites, GRINM Group Co., Ltd., No.11, Xingke East Street, Yanqi Economic Development Zone, Huairou District, Beijing 101407, China; zhs200559@163.com; 2 GRINM Metal Composites Technology Co., Ltd., No.11, Xingke East Street, Yanqi Economic Development Zone, Huairou District, Beijing 101407, China; 3 General Research Institute for Nonferrous Metals, No.2, Xinjiekouwai Street, Beijing 100088, China

**Keywords:** compound casting, numerical simulation, layered aluminum matrix composite, microstructure, mechanical properties

## Abstract

The requirements of high-strength, wear-resistance and lightweight of brake drums have been continually increasing in recent years and any specific aluminum alloy or particle-reinforced aluminum matrix composites may not satisfy all the demands. Combining dissimilar materials to play their respective advantages is a solution to this problem. In this study, a compound casting method was used to combine solid SiC_p_/A357 composite and a liquid 7050 aluminum alloy to prepare an aluminum matrix composite with a layered structure. The ProCAST numerical simulation software was used to predict the heat transfer in compound casting process and guide the preheating temperature of the wear-resistant ring in the experiment. An Optical Microscope (OM) and Scanning Electron Microscope (SEM) were used to observe microstructures around the solid–liquid bonding interface, the element distribution and phase component of which were analyzed by Energy Dispersive Spectroscopy (EDS) and mechanical properties were evaluated by microhardness and shear tests. The results showed that the interface of the layered aluminum matrix composite prepared by this method achieved complete metallurgical bonding and a transition zone formed on the solid surface. After T6 heat treatment, the average shear strength of the interface increased from 19.8 MPa to 33.8 MPa.

## 1. Introduction

Aluminum alloys are widely used in automotive and aerospace industries as lightweight materials with the greatest potential to replace steel [1,2,3]. On some key parts, due to the complex structure and special requirements of local performance, such as the brake drum in the automotive braking system, which requires a certain strength and heat dissipation performance, but also to ensure the external wear resistance, a single aluminum alloy cannot meet the performance requirements of the parts [4,5]. By combining two kinds of metal materials with different properties by special means, the layered composite material can give full play to the advantages of the two materials and overcome their disadvantages to meet the performance requirements of complex working conditions. Bimetallic layered composites have been widely used in the industrial field [6]. Welding is a common means to combine different materials, such as friction stir welding [7], gas metal arc welding [8], laser beam machining [9], etc. Rolling can also combine plate-like heterogeneous materials at certain temperatures and pressures, such as cold rolling or hot rolling [10]. Although these methods can obtain a good interface of different metal materials, their application in some composite parts is restricted by complex process and geometric shape [11,12,13].

Solid–liquid compound casting is a relatively simple and low-cost preparation process, in which the metallurgical bonding of two metals is achieved by casting the liquid material onto the solid material and a continuous metal transition zone is formed [14,15]. However, it is difficult for an aluminum alloy to bond with another material in the process of compound casting. The wettability between liquid and solid aluminum alloy is seriously affected due to the formation of a layer of alumina on the surface of solid aluminum alloy [16,17,18]. In order to solve the problem of poor wettability of aluminum alloy, many researchers plated the solid surface of aluminum alloy with a layer of metal coating that is difficult to oxidize before the solid–liquid compound casting. Among all metal coating materials, zinc is the most commonly used and the easiest to form from a continuous transition layer in the interfacial region due to its low melting point (about 420 °C), high solubility at high temperatures and its satisfactory wettability with melt aluminum [19,20,21,22,23,24,25]. Plating other metal layers on the surface of aluminum alloy is undoubtedly a good way to improve the wettability of solid and liquid. However, in the actual engineering production process, it is difficult to coat the surface of large structural parts and the uniformity of coating cannot be guaranteed. The surface coating increases the production process and cost and it is more beneficial for engineering applications to seek more stable and efficient solid–liquid bonding methods.

In a previous study by the author [26], the layered composite brake drum parts of 7050 aluminum alloy and A390 aluminum alloy were prepared by a liquid die forging compound casting method. It was found that the preheating temperature of solid and casting temperature of melt are the main factors affecting the interface bonding state. However, in the case of solid without coating, the higher the preheating temperature of the solid, the higher the degree of surface oxidation, which is not conducive to the wetting of the melt. At the same time, for 7xxx aluminum alloy, the higher the casting temperature, the larger the grain size and the worse its mechanical properties [27,28,29,30]. Therefore, it is still a challenge to obtain layered composite materials with good interfacial bonding under the premise of ensuring high strength and toughness of the matrix. Optimizing the compound casting parameters is of great significance for obtaining layered composite materials with good properties.

The Finite Element Method (FEM) has been widely studied in the field of casting [31,32,33]. Numerical simulation of casting processes can help staff to effectively predict the location, size and time of various possible defects during the casting process design stage, so as to optimize the process design, ensure casting quality, shorten the experimental period and reduce the production cost. ProCAST software (2013.0, Dayton, OH, USA), developed by UNIVERSAL ENERGY SYSTEM (UES), is mainly used to simulate the melt flow and solidification during the casting process. By establishing a finite element mathematical model and solving it through a comprehensive numerical analysis, the temperature field and stress field in the casting process are relatively accurately simulated. Many researchers have used ProCAST software to simulate the compound casting process [34,35].

In this study, the scale-reduced parts of the brake drum were taken as the research object. Under the premise of no metal coating on the solid, SiC_p_/A357 composite with excellent wear resistance [36] as the outer wear-resisting layer and 7050 aluminum alloy as the inner skeleton were used for composite casting, so that the two can be fully wetted into a metallurgical combination. The influence of different wear-resisting layer sizes and thicknesses on surface temperature change was simulated by ProCAST software. And the microstructure, morphologies and mechanical properties of the solid–liquid bonding interface were studied in combination with the experiment.

## 2. Materials and Methods

### 2.1. Materials

In order to prepare SiC_p_/A357-7050 layered composite brake drums, 15 wt.%SiC_p_/A357 composite was used as solid wear-resistant outer layer and 7050 alloy as high strength inner layer. The 15 wt.%SiC_p_/A357 composite material was prepared by stirring casting method, the average SiC particle size of which was 14 μm. The SiC_p_/A357 composite material was cast and machined into rings of a certain thickness and height. The 7050 alloy was made by smelting high purity aluminum ingot, high purity zinc ingot, high purity magnesium ingot, Al-5Zr intermediate alloy and Al-2Sc intermediate alloy. The chemical components of A357 and 7050 used in this study are listed in Table 1. The chemical composition of the alloys was measured by a floor-standing electric spark direct reading spectrometer (FOUNDARY MASTER PRO, Oxford Instruments, Abingdon, UK).

**Figure 1:**
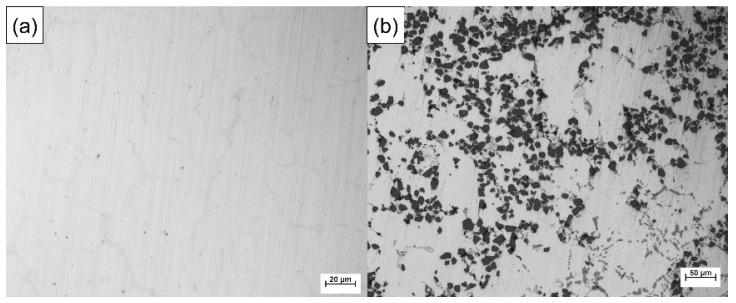
Optical metallographic diagram of 7050 alloy (**a**) and 15 wt.%SiCp/A357 composite (**b**).

Particle-reinforced aluminum matrix composites (PAMCs) have good friction resistance, but their elongation and strength are usually poor [37,38]. On the other hand, 7050 is one of the strongest aluminum alloys of the 7xxx series and will produce significant precipitation strengthening effect after T6 heat treatment, while the introduction of Sc element will significantly reduce the grain size, which can further produce the effect of grain boundary strengthening and increase its elongation [39,40]. Figure 1 shows the optical metallographic diagram of 7050 alloy and 15 wt.%SiC_p_/A357 composite. Manufacturing of PAMC/7xxx layered composites with a good metallurgical bonding interface may be a new approach to obtain a high wear resistance and high strength and toughness materials at the same time.

### 2.2. Numerical Simulation of the Compound Casting Process

**Figure 2:**
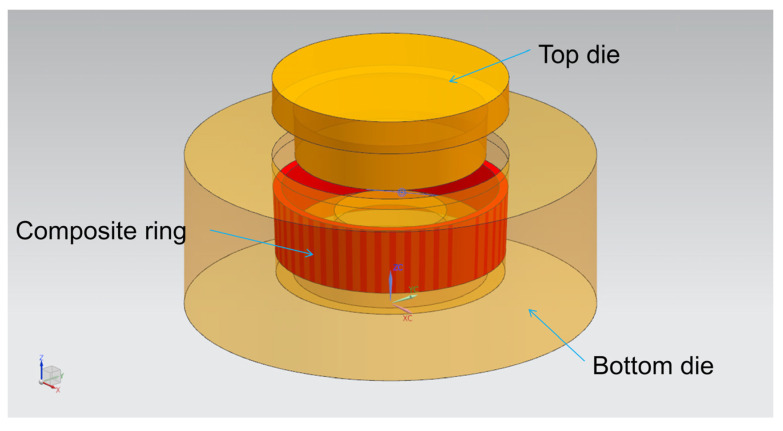
Dies assembly model for compound casting drawn by UG software.

**Figure 3:**
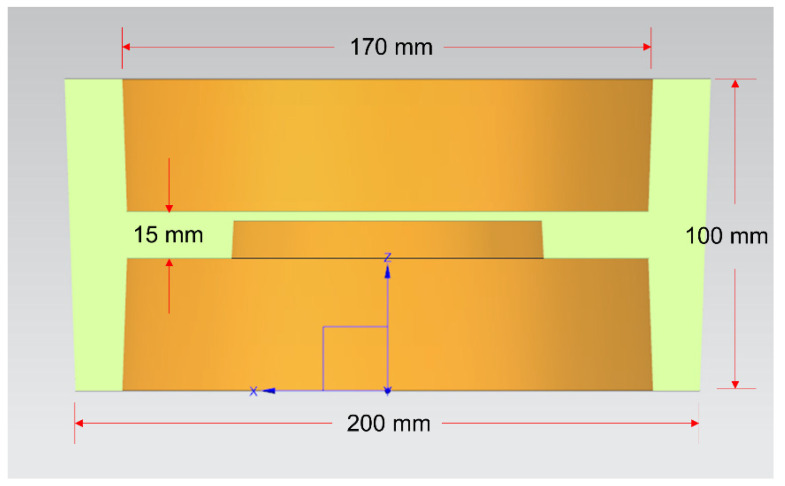
Schematic diagram of the size of the part blank profile.

The dies assembly model for compound casting is drawn by UG software (NX10.0, Siemens PLM Software, Munich, Bavaria, German), as shown in Figure 2. The cavity after the dies are completely closed is the final size of the part blank, with the outer diameter of 200 mm, inner diameter of 170 mm, height of 100 mm, the middle thickness of 15 mm and the draft angle of 2°, as shown in Figure 3.

**Figure 4:**
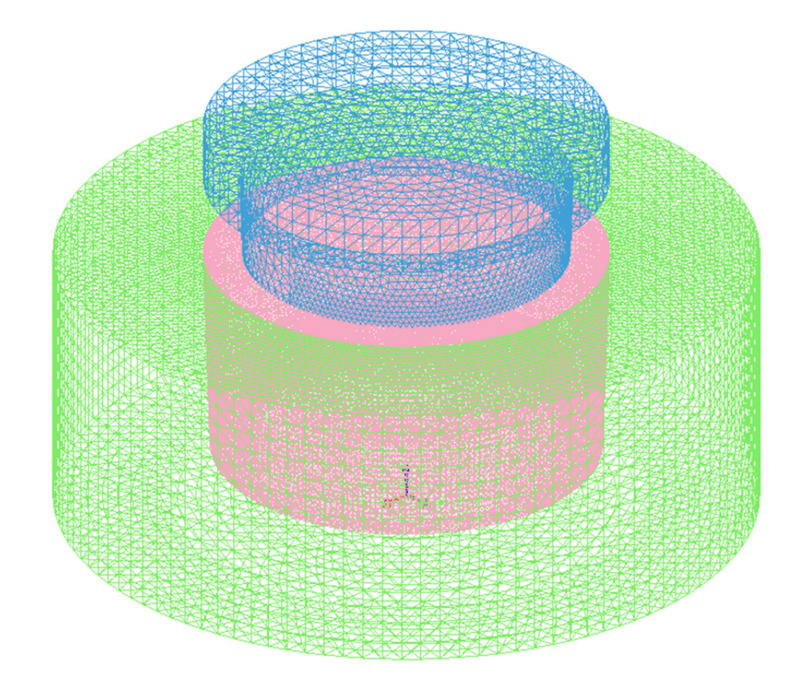
3D finite element mesh of the compound casting model.

ProCAST software was used for the numerical simulation in this study, which mainly predicted the temperature field during the compound casting process. In the Pre-processing, the gravity setting was the same as the real environment of g = 9.8 m·s^−2^ and the direction was along the -Z axis. The dies were set to the type of “Mold”, the material was “H-13 steel” and the stress form of which was set to “Rigid”. The composite ring was set to the type of “Core”, the material was “Al7Si0.4Mg”, which the influence of SiC_p_ on heat transfer performance was ignored for the convenience of calculation and the stress form of the composite ring was set to “Rigid”. The type of the metal melt was set to “Alloy”, the stress form of which was set to “Elastoplastic” and the material was 7050 alloy, which did not exist in the ProCAST materials database. In order to simulate more accurately, according to its composition, the physical performance parameters of 7050 alloy at different temperatures were automatically generated, including enthalpy curve, solid phase ratio curve, density, thermal conductivity, etc., with the help of ProCAST’s Scheil micro-segregation model. It is assumed that the solute of the melt is completely diffused in the liquid phase, but there is no diffusion in the solid phase [41,42]. The 2D (triangular type) and 3D (tetrahedral type) meshes were 56,862 and 1,042,252, respectively, as shown in Figure 4.

In the simulation process, the melt pouring process was ignored and 7050 alloy with a certain quality was placed in the cavity beforehand. In the studies of the influence of aluminum alloy liquid die forging process parameters on the mechanical properties of formed parts [43,44,45,46], it is generally believed that the casting temperature is between 650–730 °C, the mold temperature is between 150–250 °C and the specific pressure is between 50–100 MPa, which can obtain better performance. Based on the previous research, for the numerical simulation parameters of this experiment, the casting temperature was 660 °C, the solid ring preheating temperature was 25–300 °C, the die temperature was 200 °C, the specific pressure was 100 MPa and the working speed of the top die was 20 mm·s^−1^. The melt filled the cavity for about 3 s and the pressure was relieved after holding for 10 s. When the temperature of the part dropped to 400 °C, the calculation stopped.

Some studies have shown that the applied pressure will affect the interface heat transfer coefficient between the metal melt and the metal die [47,48,49,50,51]. It is generally believed that the liquid contacts the metal surface more closely after the melt is pressurized. On the microscopic level, the solid–liquid contact interface increases and the macroscopic manifestation is the increase of the interface heat transfer coefficient. However, there is currently no unified quantitative relationship between pressure and interface heat transfer coefficient. In this study, the heat transfer coefficient of the interface between the melt and the die is referred to in the research results [52] and combined with our own experimental conditions. The value of the interface heat transfer coefficient before and after the pressure is shown in Table 2. Before the melt fills the cavity, it is regarded as unpressurized. When the melt fills the cavity and stops the flow, it is regarded as pressurized.

**Figure 5:**
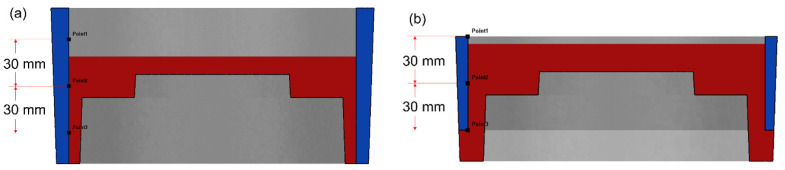
Surface temperature observation position of long ring (**a**) and short ring (**b**).

The height of the composite ring was designed to be 60 mm and 100 mm respectively, taking into account the machining allowance of the brake drum and the size of the friction required in the working conditions, called the long ring and the short ring. The draft angle of the outer side of the composite ring was 2°, the inner side had no draft angle and the thickness regardless of the draft angle was 5 mm. After the calculation, select the Y–Z section to observe the temperature change of the ring surface. The observation positions of the long ring and the short ring are shown in Figure 5. The assembly position of the short ring was the center of the mold cavity and the observation position of the long ring and the short ring were the same relative to the assembly position.

### 2.3. Manufacturing and Heat Treatment of Layered Composite Materials

**Figure 6:**
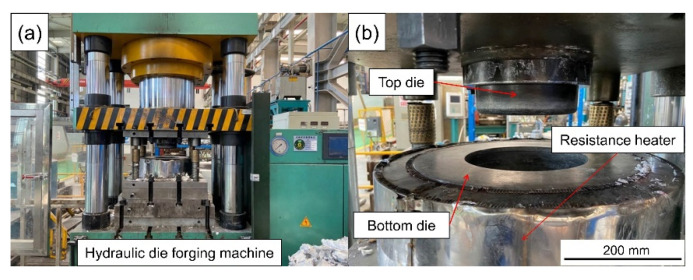
Physical diagram of hydraulic die forging machine (**a**) and die assembly (**b**).

The dies of the scale-reduced brake drum for compound casting were designed and manufactured. A resistance heating cylinder was sleeved on the outside of the mold for preheating. The draft angle of the bottom die cavity was 2°. The LYF-400SA hydraulic forging machine (Dongguan Liyuan Hydraulic Technology Co., Ltd., Dongguan, China) was used for pressurization, the maximum pressure was 400 t, the maximum pressurizing speed was 25 mm·s^−1^. The compound casting action was operated on the PLC digital display touch screen control system on the hydraulic forging machine. The actual die assembly is shown in Figure 6.

Before compound casting, in order to protect the dies and make the melt forming and part ejection proceed smoothly, it is necessary to preheat the dies and spray the release agent on the surface of the dies. The SiC_p_/A357 composite rings were strictly machined in advance according to the model size in the numerical simulation and the inner surface was subjected to alkali washing with 10 wt.% NaOH aqueous solution and then acid washing with 10 vol.% HNO_3_ aqueous solution. After removing the surface oxide layer, composite rings were placed in a vacuum drying furnace for preheating. While compound casting, the preheated SiC_p_/A357 composite ring was quickly removed from the vacuum drying furnace and placed in the die cavity as soon as possible to reduce the oxidation and then a quantitative 7050 melt was poured into the cavity, the top die went down so that the liquid 7050 alloy was combined with the solid SiC_p_/A357 composite under pressure. After full solidification and cooling, the molded part was ejected and removed, thereby a layered composite brake drum was manufactured.

The outer layer of the composite brake drum bears the wear-resistant function and the inner layer bears the high-strength structural function. The parts need to be put into use after heat treatment. The heat treatment procedure of 7050 alloy and A357 alloy is different and the solution temperature of the latter is higher than that of 7050 alloy. In order to avoid overheat, the heat treatment procedure of composite part should be adopted to that of 7050 alloy. A large number of studies on the heat treatment of 7xxx series aluminum alloys show that the eutectic point of 7xxx series aluminum alloys is 477 °C and the solid solution temperature is generally about 470 °C [53,54,55,56]. The T6 heat treatment procedure used in this experiment was 450 °C × 2 h + 460 °C × 2 h + 470 °C × 2 h + 120 °C × 24 h. The solution treatment was carried out in a box-type resistance furnace and the aging treatment was carried out in an air blast drying box.

### 2.4. Microstructure Observation and Mechanical Properties Characterization of Bonding Interface

**Figure 7:**
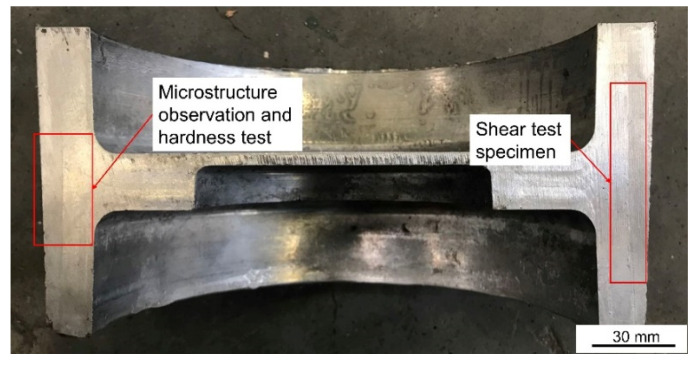
Physical diagram of cross-section of the brake drum and its sampling position and function.

The sampling location and its function are shown in Figure 7. Optical microscope (OM) and Scanning electron microscope (SEM) were used to observe the microstructure of the part and the phase distribution and element composition were analyzed by energy dispersive spectroscopy (EDS). Before OM and SEM observation and EDS analysis, the observation surface was polished to achieve a mirror finish. Zeiss Axiovert 200 MAT OM (Zeiss, Oberkochen, Germany) was used for optical metallographic observation, the higher magnification microstructure was observed by field emission SEM (JSM-7900F, JEOL Ltd., Tokyo, Japan) and the element composition and distribution of the observation area were analyzed by EDS. The Vickers hardness testing machine (Wilson VH1150, Buehler, Lake Bluff, IL, USA) was used to test the microhardness within 400 μm on both sides of the bonding interface with a test interval of 50 μm. A total of three groups were tested and the average value was taken. The experimental load was 0.5 kg and the holding time is 10 s.

**Figure 8:**
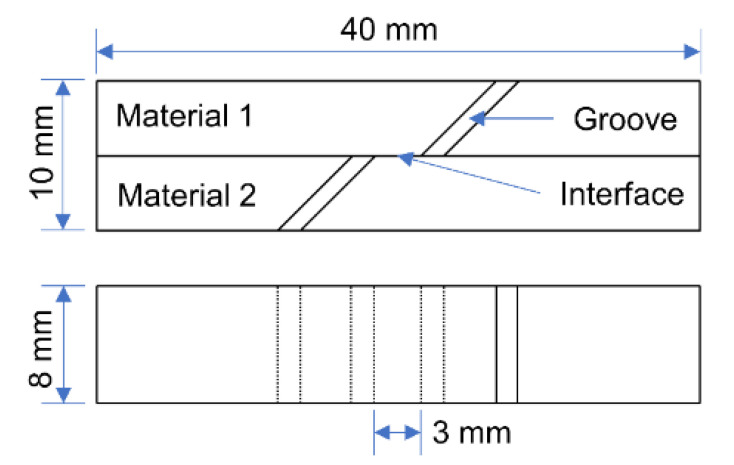
Schematic diagram of shear test specimen.

The interface bonding strength is characterized by the shear strength. The schematic diagram of the shear test specimen is shown in Figure 8. The shear tests were performed at a constant tensile rate of 2 mm·min^−1^ on a WDW-200 electronic universal testing machine. When the specimen is tensed, the bonding interface in the middle of the specimen is subjected to shearing stress. When the load exceeds the binding force, the bonding interface of the specimen will separate and fracture. At this time, the shear strength can be calculated by Equation (1).

τ = P/wL(1)

where τ is the interfacial shear strength, in Mpa; P is the failure load, the unit is N; w is the sample width, unit is mm; L is the interface lap length, unit is mm. In this experiment, w = 8 mm, L = 10 mm.

## 3. Results and Discussion

### 3.1. Heat Transfer Simulation and Experimental Verification during Compound Casting

**Figure 9:**
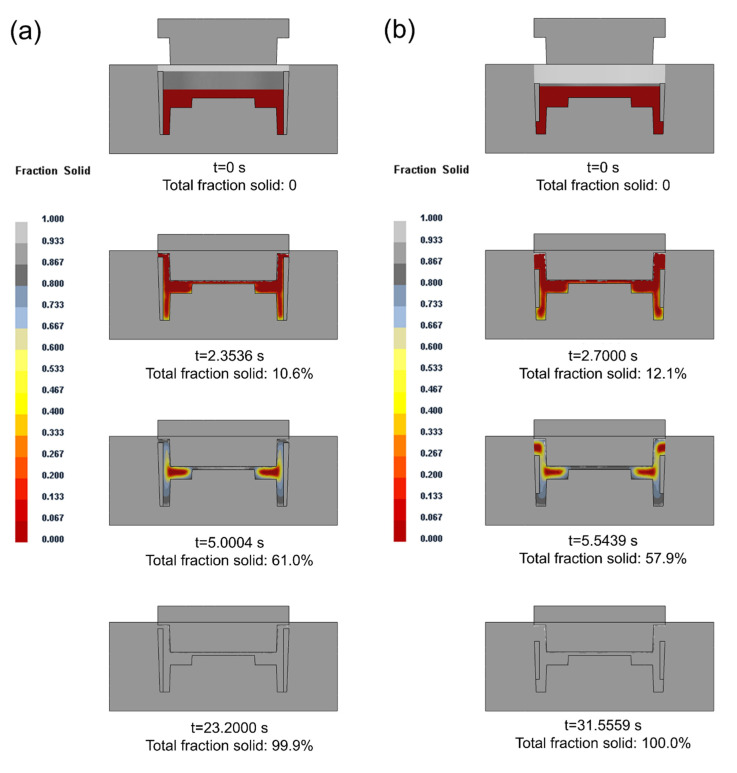
Change of solid fraction in the compound casting process of long ring (**a**) and short ring (**b**).

When the preheating temperature of the composite ring was 200 °C and the casting temperature was 660 °C, the change of solid fraction in the compound casting process is shown in Figure 9. In the solidification process, the solidification time of the long ring was 23.2000 s, less than 31.5559 s when the composite ring was short. When the composite ring was short, the solid volume decreased, which actually increased the liquid–solid ratio. The amount of liquid to be solidified increased, so the solidification time became longer. It can be seen from the results that when the composite ring was short, two hot spots appeared in the casting, respectively in the middle and top of the rim of the brake drum.

**Figure 10:**
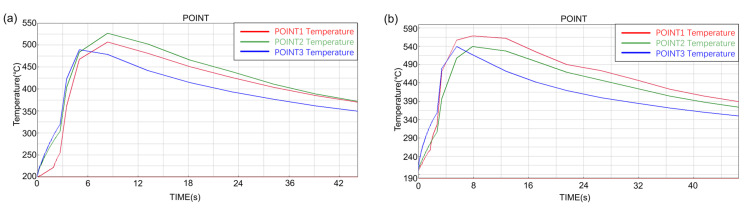
The temperature–time curve of the inner surface of long ring (**a**) and short ring (**b**).

The temperature–time curve of the inner surface of the composite ring is shown in Figure 10. The three observation points 1, 2 and 3 correspond to the positions of the top, middle and bottom of the inner surface of the composite ring respectively, as shown in Figure 5. The temperature of the middle part of the long ring increased the most, while that of the top part of the short ring increased the most. Comparing the maximum temperature rise values at different observation points, the maximum temperature rise values at different observation points were different. It can be seen from the curve that the maximum temperature rises values at different locations of the long ring differed by about 35 °C, while that at different locations of the short ring differed by about 27 °C. The temperature change at the three observation points of the short ring was more uniform than that of the long ring.

**Figure 11:**
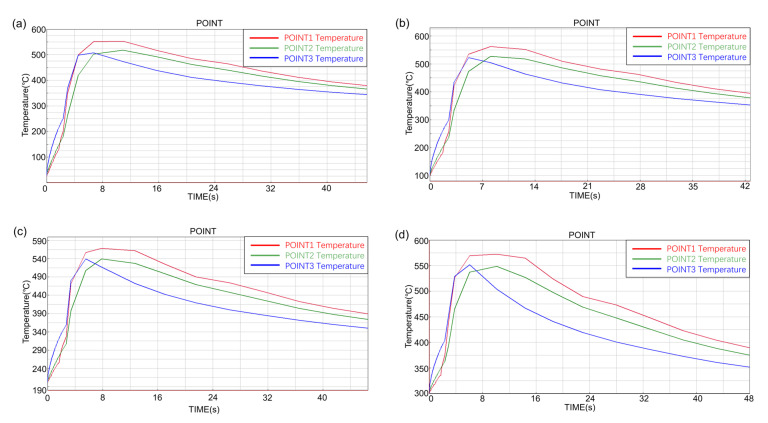
The temperature–time curve of the inner surface of short ring at different preheating temperatures: (**a**) 25 °C, (**b**) 100 °C, (**c**) 200 °C and (**d**) 300 °C.

Observe the inner surface temperature rise of the short ring at different preheating temperatures, as shown in Figure 11. It can be seen from the results that the inner surface temperature of the short ring with different preheating temperatures had the same rising trend, the bottom temperature rose rapidly and the maximum temperature was almost equal to the middle temperature. The top temperature rose faster than the middle and the highest temperature was higher than the rest.

**Figure 12:**
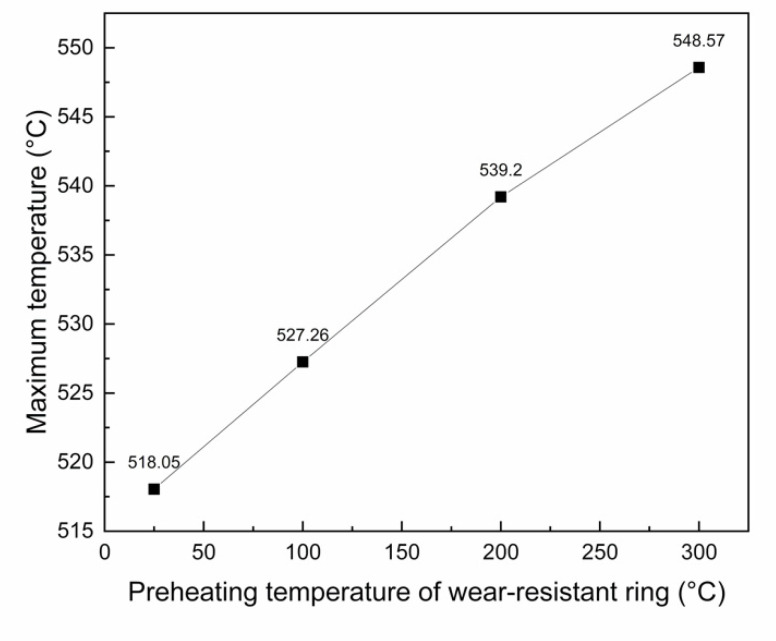
Relationship between the maximum temperature of the inner surface of wear-resistant ring and the preheating temperature.

Considering the relatively uniform temperature rise of the short ring and the shear stress of the brake drum mainly concentrated at the junction of spokes and rims, the corresponding position is the interfacial bonding area in the middle of the wear-resistant ring, so the interfacial bonding situation in the middle will be mainly considered in the future. The maximum temperature rise in the middle of the inner surface of the wear-resistant ring was taken for comparison, as shown in Figure 12. With the increase of the preheating temperature of the wear-resistant ring, the maximum temperature of the inner surface also increased and the relation with the preheating temperature of the wear-resistant ring was almost linear.

**Figure 13:**
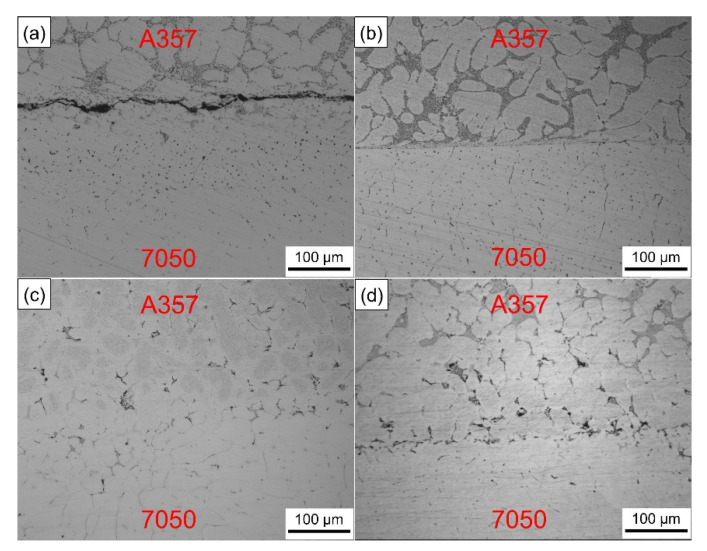
Interfacial metallographic structure of A357 ring and 7050 compound casting parts at different preheating temperatures: (**a**) 25 °C, (**b**) 100 °C, (**c**) 200 °C and (**d**) 300 °C.

The numerical simulation results reflect the temperature variation trend of the inner surface of the wear-resistant ring and the influence of preheating temperature on the solid–liquid bonding was verified through experiments. Figure 13 shows the interfacial metallographic structure of A357 alloy ring and 7050 alloy compound casting parts at different preheating temperatures. When the preheating temperature of the wear-resistant ring was 25 °C, it can be seen that there were large gaps between the solid–liquid bonding interface. In the process of compound casting, the temperature of the ring was not enough to rise above the solidus (550 °C). The melt solidified as soon as it touched the solid surface, wetting and element and diffusion could not take place effectively. When the preheating temperature of the ring was 100 °C, the A357 microstructure was almost unchanged. The A357 ring and the 7050 melt mainly rely on the wetting and limited diffusion of surface elements to form the bonding, which fails to achieve good metallurgical bonding. When the preheating temperature of the ring was 200 °C, it can be found that the microstructure of the A357 side changed greatly, the grain spheroidized, the eutectic silicon phase decreased and new phases were formed. The interface between A357 and 7050 became blurred and discontinuous, and could only be distinguished by the different phase morphology, which can be considered as good metallurgical bonding. When the preheating temperature of the ring was 300 °C, the interface bonding was not as good as that when the preheating temperature of the ring was 200 °C. However, compared with the low preheating temperature, the microstructure of the A357 side still changed, the grain spheroidization occurred and the interface was obvious and flat.

**Figure 14:**
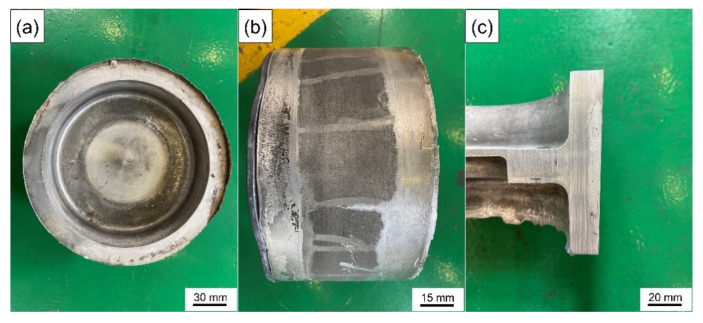
The actual image of the brake drum: (**a**) top view, (**b**) side view (The iron gasket with a certain thickness is to facilitate ejection and demolding) and (**c**) section view.

Although the high preheating temperature can increase the wettability between the solid metal and the liquid melt, the high temperature is easy to oxidize the surface of the solid metal [57], which seriously hinders the wetting between the melt and the metal matrix and causes the deterioration of the interface. Aluminum alloy is easy to oxidize in air and is sensitive to temperature. The oxide film produced is compact and continuous and is not easy to damage or penetrate. Even after chemical descaling treatment, the secondary oxidation caused by sudden temperature rise in the casting process cannot be avoided. Therefore, according to these results, the suitable preheating temperature of the wear-resistant ring should be 200 °C. Figure 14 is the actual image of the brake drum manufactured by compound casting.

### 3.2. Microstructure of Solid–Liquid Bonding Interface

**Figure 15:**
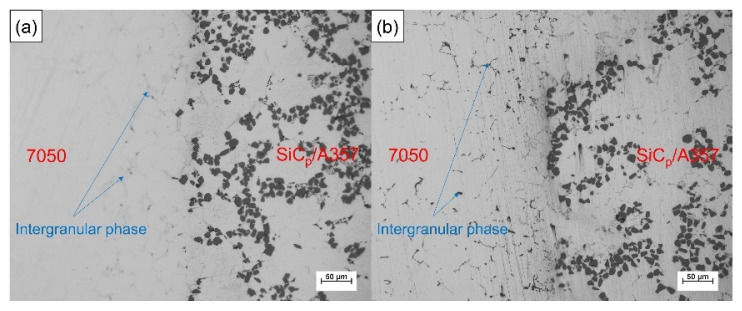
Optical metallography of the solid–liquid bonding interface in as-cast (**a**) and T6 (**b**) status.

SiC_p_/A357 wear-resistant ring with preheating temperature of 200 °C and 7050 alloy melt were compound-cast at 660 °C and specific pressure of 100 MPa. The optical metallography of the solid–liquid bonding interface is shown in Figure 15, where (a) is the interface microstructure in as-cast status and (b) is the interface microstructure in T6 status. The solid–liquid interface bonding was good and there were no visible gaps and cracks, thus achieving complete metallurgical bonding. Enrichment of intergranular phases occurs beside the interface, as indicated by the arrow in the graph. In the as-cast microstructure, the intergranular phase was gray, while after T6 heat treatment, the intergranular phase became black, indicating that the new phase was formed during the heat treatment. It can be seen from Figure 1 that this phase is neither the common phase of 7050 alloy nor the phase that may be formed in A357 alloy, and it is necessary to further explore and determine its composition.

**Figure 16:**
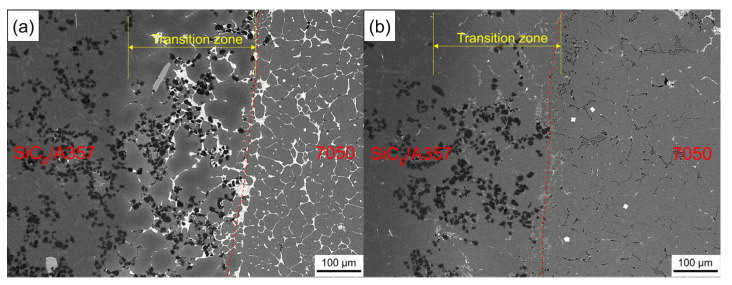
SEM backscattered photograph of the solid–liquid bonding interface in as-cast (**a**) and T6 (**b**) status.

Figure 16 shows the SEM backscattering of the solid–liquid bonding interface of the layered composite. The solid–liquid bonding interface of the as-cast sample had a diffusion layer of about 200 μm. Different from the original microstructure, in the transition zone, bright white phase appeared and was distributed among α-Al grains accompanied with SiC_p_. After T6 heat treatment, the bright white phase in the transition zone disappeared and a gray phase appeared, while a black intergranular phase appeared in the 7050 side, which was consistent with the optical photograph, as shown in Figure 15.

**Figure 17:**
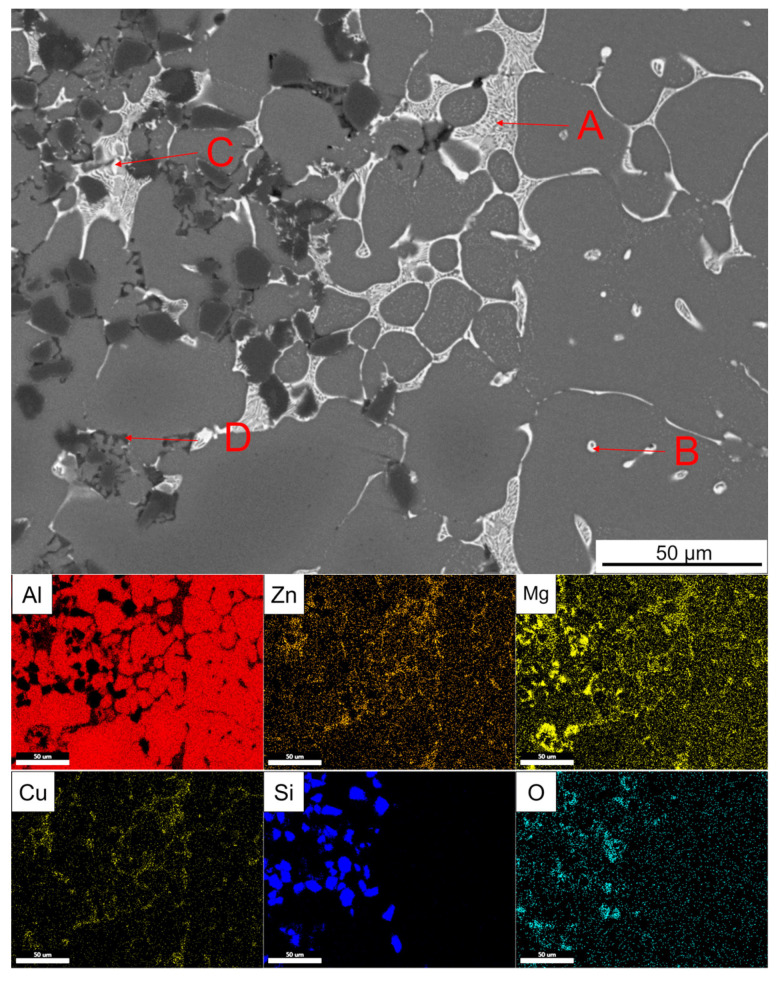
SEM backscattered photograph of an enlarged part of the solid–liquid bonding interface in as-cast status and its EDS surface scanning results. (**A**–**D** are the EDS scanning points).

Figure 17 shows the enlarged part of the solid–liquid bonding interface in the as-cast status and EDS surface scanning results. It can be seen that bright white reticular phase and point-like phase were mainly distributed on the 7050 side, while bright white reticular phase and a black coarser reticular phase are mainly distributed on the SiC_p_/A357 side. The EDS point scanning results are shown in Table 3. AlZnMgCu phase was the dominant intergranular phase on the 7050 side and a very small amount of the Fe-containing phase was associated with the AlZnMgCu phase. Bright white AlZnMgCu phase also existed in the transition zone on the SiC_p_/A357 side, indicating that remelting occurred on the solid surface during the solid–liquid bonding process. Elements Zn, Cu and Mg in 7050 alloy diffused into SiC_p_/A357 to form an AlZnMgCu phase. The content of Mg and O elements in the black reticular phase on the SiC_p_/A357 side was high. Within the range of error, it is speculated that the oxide layer on the solid surface reacted with the melt. According to the study [58], the Mg element has a great affinity to the O element, which can deprive oxygen element to generate MgAl_2_O_4_ and promote the interface wetting.

**Figure 18:**
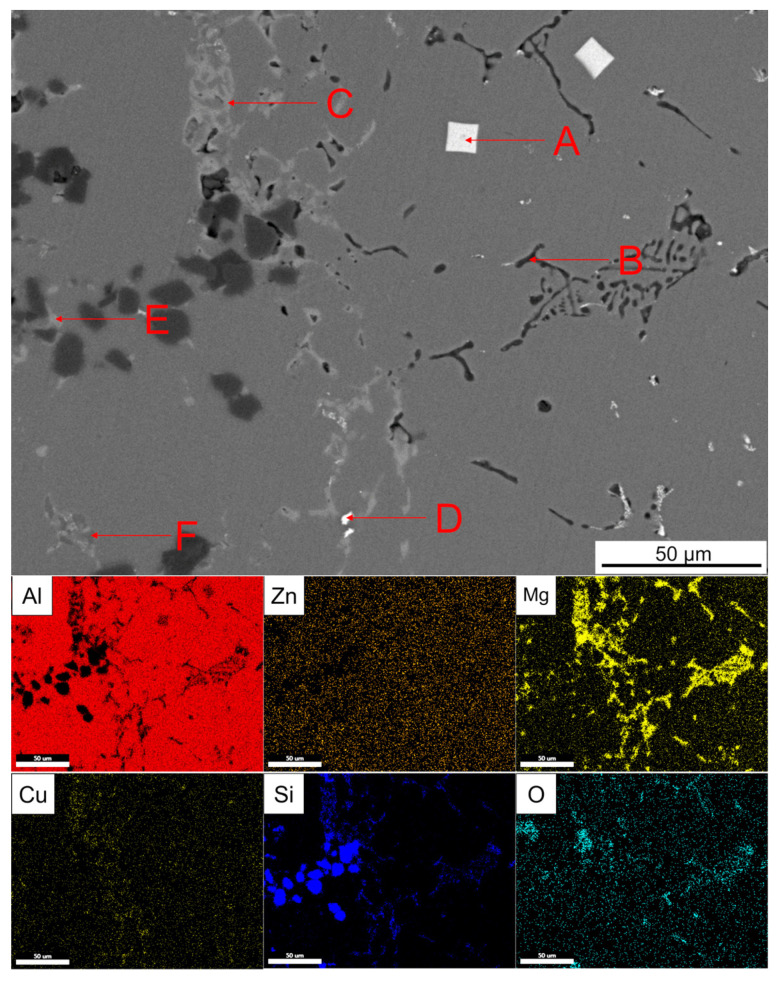
SEM backscattered photograph of an enlarged part of solid–liquid bonding interface in T6 status and its EDS surface scanning results. (**A**–**F** are the EDS scanning points).

Figure 18 shows the enlarged part of the solid–liquid bonding interface in as-cast status and EDS surface scanning results. It can be seen that black reticular phase and the bright white square phase and dot phase were mainly distributed on the 7050 side. The grey phase was distributed at the solid–liquid bonding interface. The bright white reticular phase disappeared in the transition zone on the SiC_p_/A357 side and a small amount of slight gray and dark gray phases existed. The T6 heat treatment made the AlZnMgCu phase in the as-cast microstructure disappear and the Zn element uniformly distributed in the whole observation surface, leaving the phases with high melting point at the solid–liquid bonding interface, compared with Figure 17. The EDS point scanning results are shown in Table 4. On the 7050 side, the primary phases of Al_3_(Zr_x_, Sc_1−x_) with a regular tetragonal shape can be seen, which could be used as nucleating particles to refine grain. The place where there was originally an AlZnMgCu phase between the grains was replaced by a Si-containing phase, O element also diffused to the 7050 side with the Si element, according to Figure 18. The solid–liquid bonding interface was a Cu-rich region. With the diffusion of the Si element in A357 alloy, the element Zn in the AlZnMgCu phase originally enriched at the interface was replaced by a Si element to generate the AlCuMgSi phase, which might be the W quaternary phase. The AlZnMgCu phase of the transition zone on the SiC_p_/A357 side was also replaced by an AlCuMgSi phase, while the excess Si elements in A357 still existed in the form of eutectic silicon.

According to the compound casting parameters guided by numerical simulation, SiC_p_/A357 composite was well bonded with the 7050 melt and remelting occurred on the surface of SiC_p_/A357 composite, forming absolute metallurgical bonding. Solute elements in the 7050 melt were enriched at the solid–liquid bonding interface and continued to diffuse to the SiC_p_/A357 side for about 200 μm, forming an obvious transition zone. In the compound casting process, due to the ability of the Mg element to capture the O element, the oxidation film of the solid surface, which prevented the liquid from wetting the solid was efficiently avoided, so that the 7050 melt could better contact SiC_p_/A357. After T6 heat treatment, the Zn element uniformly diffused to the entire observation surface, while the Cu element in 7050 alloy and the Si in A357 alloy mutually diffused to the opposite side, generating new phases that did not exist in the original microstructure at both sides of the interface.

### 3.3. Mechanical Properties

**Figure 19:**
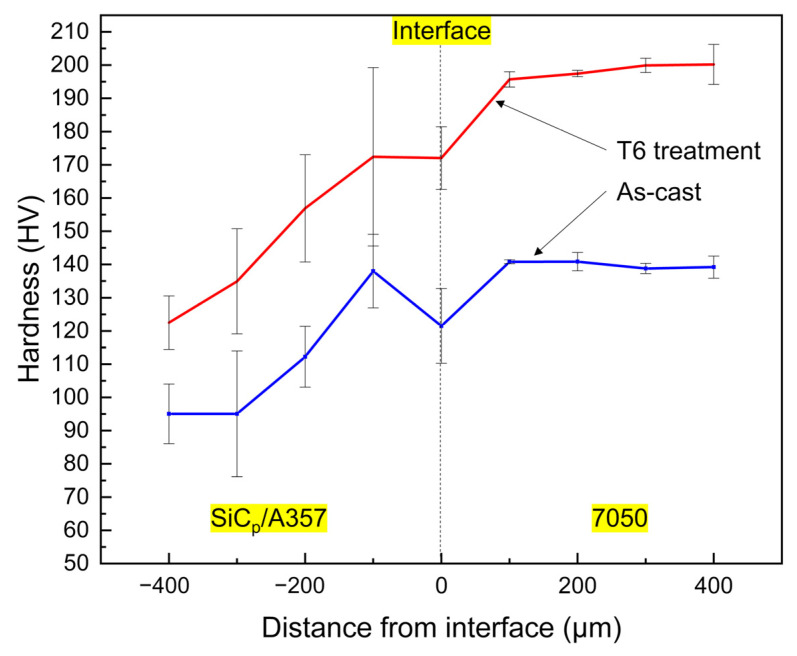
Variation of hardness across the solid–liquid bonding interface.

The continuous variation of hardness in the range of 400 μm on both sides of the interface is shown in Figure 19. The hardness on the SiC_p_/A357 side increased as it approached the interface, while the hardness on the 7050 side decreased as it approached the interface. No matter if it is as-cast status or T6 status, the hardness variation on both sides of the interface was relatively continuous, without obvious hardness mutation, indicating that no serious intermetallic compound layer aggregation occurs at the interface, which is consistent with the element distribution in Figure 17 and Figure 18.

As for the as-cast sample, the alloying degree of the SiC_p_/A357 matrix near the interface was increased due to the diffusion of solute elements from the 7050 alloy melt into SiC_p_/A357 and the alloying degree was inversely proportional to the distance from the solid–liquid bonding interface. A large number of Zn, Mg and Cu elements formed hardening phase such as AlZnMgCu and Mg_2_Si, as shown in Figure 17, which lead to the hardness of SiC_p_/A357 near the interface having a certain degree of improvement compared with the original SiC_p_/A357 composite, while the hardness change on the 7050 side was not that obvious.

**Figure 20:**
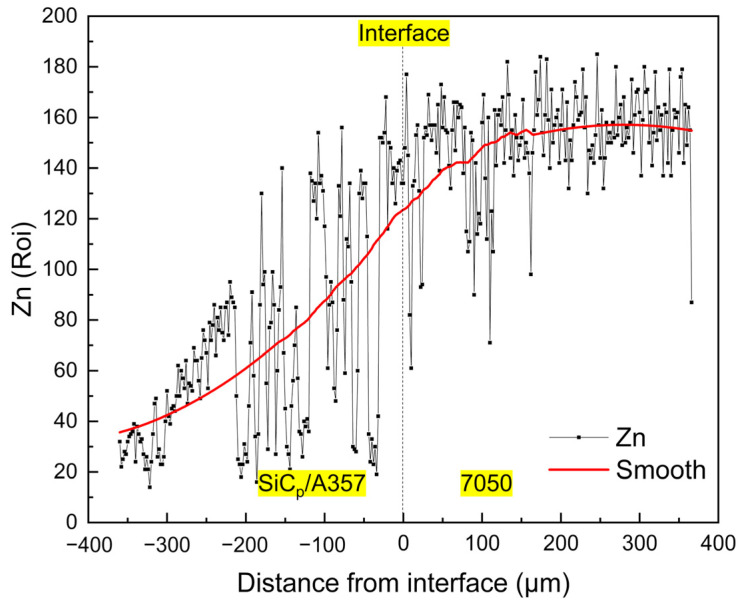
Concentration variation of Zn element across the solid–liquid bonding interface in T6 status.

After T6 heat treatment, the solute elements were homogenized, the AlZnMgCu phase with a lower melting point disappeared during the solution process and the phase with the higher melting point was left or formed due to the diffusion and migration of the elements. During the aging process, the supersaturated solid solution precipitates out a finer nanophase [59,60,61], resulting in an increase in hardness. As for this kind of solid–liquid bonding interface, the main cause of hardening is precipitation strengthening and the nano-precipitated phase that can produce precipitation strengthening is mainly an η’ (MgZn2) phase and the increase in hardness is significantly related to the fraction of the η’ phase [62,63,64,65]. During the T6 heat treatment process, the Mg and Zn in 7050 further diffused to the low concentration region, resulting in continuous variation in the concentration of solute elements on both sides of the interface, making the concentration of solute on the 7050 side of the interface lower than that of the 7050 alloy matrix. Figure 20 shows the distribution of the Zn element at the interface after T6 heat treatment. Obviously, the concentration of Zn element is positively correlated with the content of the η’ phase and the concentration of the Zn element across the interface is very consistent with the variation trend of hardness, as shown in Figure 19.

**Figure 21:**
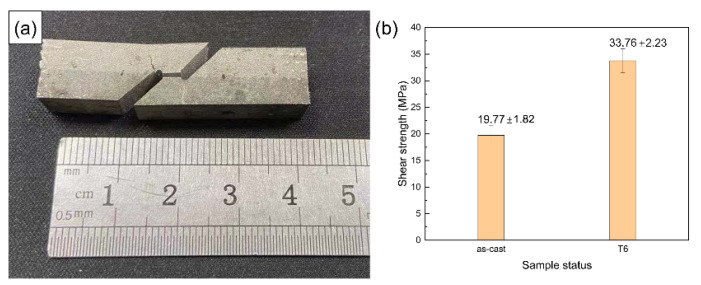
Macrograph of fractured shear test specimen (**a**) and shear strength of the solid–liquid bonding interface (**b**).

Figure 21a shows the macrograph of a fractured shear test specimen. It can be found that fracture occurred from the solid–liquid bonding interface. Therefore, the shear strength of this layered composite material depends on the firmness of the solid–liquid bonding interface. In this shear test, only the test force at fracture was recorded to calculate the shear strength and the strain was not considered. Figure 21b shows the comparison of the shear strength of the as-cast and T6 samples.

The solid–liquid compound casting method used in this experiment is low-cost and high-efficiency and large-volume layered aluminum matrix composite brake drums were prepared and the solid–liquid bonding interface can maintain a certain shear strength. After T6 heat treatment, the alloy solute elements on both sides of the interface were fully diffused, the hardness of the material was greatly improved and the interface shear strength was further improved. In future work, processing some structure pattern in the wear-resistant ring can be considered, which can form a mechanical occlusion under the premise of absolute metallurgical bonding in compound casting and further improve the bonding force of the solid–liquid bonding interface.

## 4. Conclusions

1. The liquid die forging compound casting method is a high-efficiency near-net-shape method for manufacturing large-volume layered aluminum matrix composites, without resort for coating on the solid surface, which effectively saves production cycle and cost. Under the die pressure of 100 MPa, a 7050 aluminum alloy casting temperature of 660 °C and a wear-resistant ring preheating temperature of 200 °C, only by removing the solid surface oxide layer instead of the surface coating can a complete metallurgical bonding of solid–liquid bonding interface be obtained.
2. PROCAST numerical simulation predicts that the short ring had a more uniform temperature increase than the long ring during compound casting process. The experiment proved that the wear-resistant ring preheating temperature of 200 °C was beneficial to obtain the metallurgical bonding interface without serious oxidation of the solid surface.
3. The compound casting process will cause the wear-resistant ring to produce a transition zone due to the remelting of the solid surface and the diffusion of solute elements in the melt, in which new phases will be formed, so that 7050 and SiC_p_/A357 formed a complete metallurgical bonding.
4. The layered composite material mainly relies on heat treatment precipitation strengthening to produce hardening and the hardness variation across the interface is highly consistent with the variation trend of Zn element content. After T6 heat treatment, the solute elements on both sides of the interface mutually diffuse, which increases the average shear strength of the interface from 19.8 MPa to 33.8 MPa.

## Figures and Tables

**Table 1:** Composition of the matrix alloys for composite casting (wt.%).

Alloy	Si	Cu	Mg	Mn	Fe	Zn	Ti	Zr	Sc	Al
A357	7.00	0.02	0.54	0.02	0.07	0.05	0.11	-	-	Bal.
7050	0.0001	2.4	2.3	0.0002	0.0003	6.54	0.05	0.123	0.125	Bal.

**Table 2:** Interfacial heat transfer coefficients before and after applying external pressure.

Interface	Heat Transfer Coefficient/(W·m^−2^·K^−1^)	
	Before Applying Pressure	After Applying Pressure
Metal melt/dies	2000	11,500
Metal melt/composite ring	2000	11,500
Dies/composite ring	1000	1000
Dies/air	20	20

**Table 3:** EDS analysis of each point indicated by the arrow in Figure 17 (at.%).

Point	Al	Zn	Mg	Cu	Fe	Si	O	Possible Phase
A	61.19	10.74	18.82	9.25	-	-	-	T (AlZnMgCu)
B	64.39	7.31	10.15	5.51	1.06	-	-	T (AlZnMgCu) + N (Al_7_Cu_2_Fe)
C	55.51	4.23	22.87	16.48	-	0.90	-	T (AlZnMgCu)
D	56.95	1.64	10.50	0.68	-	2.75	27.47	MgAl_2_O_4_ + Mg_2_Si

**Table 4:** EDS analysis of each point indicated by the arrow in Figure 18 (at.%).

Point	Al	Zn	Mg	Cu	Fe	Si	O	Zr	Sc	Possible Phase
A	79.77	2.08	1.49	-	-	-	-	11.34	5.31	Al_3_(Zr_x_, Sc_1−x_)
B	28.05	0.94	27.40	0.58	-	18.29	24.75	-	-	MgAl_2_O_4_ + Mg_2_Si
C	44.42	1.11	26.23	5.04		23.21	-	-	-	W (AlCuMgSi)
D	67.83	3.62	4.43	21.67	0.45	-	-	0.49	1.52	θ (Al_2_Cu) + Al_3_(Zr_x_, Sc_1−x_) + N (Al_7_Cu_2_Fe)
E	41.05	0.90	26.03	6.11	-	23.98	-	-	-	W (AlCuMgSi)
F	26.38	0.98	2.03	-	-	70.61	-	-	-	Eutectic Si

## Data Availability

Data is contained within the article.
